# Valorization of Cellulose-Based Materials from Agricultural Waste: Comparison between Sugarcane Bagasse and Rice Straw

**DOI:** 10.3390/polym15153190

**Published:** 2023-07-27

**Authors:** Wiriya Thongsomboon, Yodthong Baimark, Prasong Srihanam

**Affiliations:** Biodegradable Polymers Research Unit, Department of Chemistry and Centre of Excellence for Innovation in Chemistry, Faculty of Science, Mahasarakham University, Mahasarakham 44150, Thailand; wiriya.t@msu.ac.th (W.T.); yodthong.b@msu.ac.th (Y.B.)

**Keywords:** cellulose, sugarcane bagasse, rice straw, form construction, agricultural waste

## Abstract

Sugarcane bagasse and rice straw are major agricultural byproducts often discarded or burned as waste after cultivation, leaving their untapped potential for utilization. In this work, cellulose fibers were extracted from sugarcane bagasse and rice straw using a simple procedure: alkaline treatment with sodium hydroxide, bleaching with sodium hypochlorite, and acid hydrolysis. The obtained cellulosic materials were successfully prepared into milky white and transparent films, of which the transparency slightly decreased with the addition of glycerol. The surface of all the films appeared homogeneous with a random orientation of fibers. The rice-straw (RS) film had a more fragile texture than the sugarcane-bagasse (SBG) film. The FTIR analysis clearly indicated the functional groups of cellulose, as well as glycerol for the films mixed with glycerol. Thermal analysis showed that the native SBG film decomposed at 346 °C, higher than the native RS film (339 °C). The presence of glycerol in the films resulted in slightly lower maximum decomposition temperature (T*_d,max_*) values as well as mechanical properties. Regarding water susceptibility, the RS film had a higher percentage than the native SBG and glycerol-mixed SBG films. The extracted cellulose from both sources could form almost spherical-shaped cellulose particles. Thus, through the simple extraction method, sugarcane bagasse and rice straw could serve as excellent sources of cellulose materials for preparing cellulose films and particles, which would be advantageous to the development of cellulose-based materials.

## 1. Introduction

Biodegradable polymers have been widely studied as potential substitutes for nonbiodegradable plastics as the global plastic-waste issue escalated. Cellulose is one of the most attractive biomaterials due to its abundance and inexhaustible resources [1]. The chemical structure of cellulose is based on linear polymeric chains of D-glucose units linked by β-1,4 glycosidic bonds, with a strong hydrogen bonding network resulting in crystal structures [2]. Cellulose can be prepared into multiple scales from the nano to the micro level due to its inherent self-assembly characteristics [3]. Cellulose fibers are typically isolated from natural plants whereas lignocellulosic fibers typically comprise cellulose microfibrils tightly embedded in lignin and hemicellulose, forming rigid structures. Therefore, the pretreatment step is often used to facilitate cellulose extraction. Pretreatments reported in previous studies include alkali, carboxymethylation, enzymatic treatments, 2,2,6,6-tetramethylpiperidine-1-oxyl (TEMPO) oxidation, and acetylation [3], as well as catalytic sulfation using ion-exchange resins [4]. In addition, a mechanical process is usually performed after chemical treatments to obtain desirable cellulose fibers. Cellulose has been of interest in a wide range of applications due to various advantageous properties, particularly low cost, high strength, biodegradability, lightweight, and sustainability [5]. Cellulose-based materials have been applied in many fields, such as food packaging [6,7,8], reinforcement materials [9], coating materials [10,11], wastewater treatment [12], biodegradable packaging, flexible optoelectronics, and lightweight automobiles [13]. Cellulose, the most abundant biopolymer, is primarily derived from plants, of which various parts such as reeds, grasses, stalks, and woody vegetation are important sources of cellulose [3,14,15]. In addition, agro-industrial or agricultural wastes have been used as secondary bioresources to extract cellulose [16,17,18], including sugarcane bagasse [19,20,21] and rice straw [22,23,24].

Among the economic crops, rice is found in all regions around the world and has the largest cultivation. As rice straw accounts for approximately 45% of the volume of rice production, it is the most abundant agricultural byproduct worldwide [25,26]. Thailand is one of the biggest rice producers. Generally, most of the rice straw in Thailand has always been discarded, burned up, or used as fertilizer and animal feed. In addition, it has been used as low efficient fuel [27]. Therefore, a large amount of residual rice straw remains untapped. Rice straw shows great promise as a sustainable resource for cellulose as it is composed of cellulose (32–47%), hemicellulose (19–31.6%), lignin (11–24%), and silica (7–20%) [25,28]. To date, there have been a few studies that reported the isolation and utilization of cellulose from rice straw [29]. The rice straw-derived cellulose has been applied as absorbent fibers [30] and biocomposite reinforcement [31,32,33].

Sugarcane is also an important economic crop, the same as rice. Thailand is one of the world’s largest sugarcane producers [20]. It was speculated that the production of sugarcane worldwide will exceed 2.21 billion tons by 2024 [21]. Thus, residual sugarcane or bagasse would be produced in large quantities, reaching 280 million tons annually [34]. This byproduct is typically burned as waste in the fields, causing air pollution, emitting greenhouse gases, and impacting respiratory health [20]. The sugarcane bagasse was commonly used as a material for paper production, fermentation, and electric generation [35]. In recent years, many efforts have been made to produce high-value-added products from sugarcane bagasse. As sugarcane bagasse is composed of 40–50% cellulose, it is one of the low-cost and sustainable sources of cellulose [19,20,36]. The isolated cellulose from sugarcane bagasse has been used as a reinforcing agent in high-performance composite materials [37,38].

The northeast of Thailand is well known as the largest agricultural region for rice and sugarcane cultivation in the country. Tremendous amounts of rice straw and sugarcane bagasse remain as agricultural waste left for potential utilization. However, the extraction and utilization of cellulose from agricultural waste in this region have received limited research attention, particularly regarding sugarcane bagasse and rice straw. Therefore, this study aimed to extract cellulose from rice straw and sugarcane bagasse via simple chemical treatments to use as the raw material for film preparation. The physicochemical properties of the films made from cellulose derived from sugarcane bagasse (SBG) and rice straw (RS) were determined and compared. The morphology of the film was investigated using scanning electron microscopy (SEM). The functional groups of the films were examined by FT-IR spectroscopy. The thermal stability of the films was studied by thermogravimetric analysis. Moreover, the optical transparency and water solubility measurements were also determined. In addition to the cellulose films, cellulose particles were constructed from the extracted cellulose from both sources, and their properties were also determined and discussed. The data acquired in this work would facilitate the future development of cellulose-based materials, especially in the form of films and particles for a variety of purposes.

## 2. Materials and Methods

### 2.1. Materials

Sugarcane (*Saccharum officinarum* L.) bagasse and rice straw were obtained as general waste from a local sugarcane juice store and the local rice fields in Maha Sarakham province in the northeast of Thailand, respectively. Sodium hydroxide (NaOH), sodium hypochlorite (NaClO), sulfuric acid (H_2_SO_4_), and hydrochloric acid (HCl) were purchased from Kemaus (New South Wales, Australia), LOBA CHEMIE PVT. Ltd. (Maharashtra, India), Merck Life Science Private Ltd. (Maharashtra, India), and Ajax Finechem (New South Wales, Australia), respectively. All chemicals were reagent grade and used as received without further purification.

### 2.2. Extraction of Cellulose from Sugarcane Bagasse

The sugarcane bagasse used as the raw material for cellulose extraction was prepared by separating the hard shell and the soft inner part. The soft part was washed, dried, and crushed to obtain small fiber sizes. Cellulose from sugarcane bagasse was extracted by following the previous reports [36,37] with some modifications adjusting reagent concentrations. The prepared samples were firstly soaked in hexane for 3 h to remove wax and other residues before drying overnight at room temperature. The dewaxed sugarcane bagasse samples were cut into fine pieces before boiling (1 g/10 mL) in an alkaline solution of 7.5% (*w*/*v*) NaOH for 4 h at 80 °C to remove lignin, hemicelluloses, and other organic complexes from the samples. After washing several times with distilled water continuously to obtain neutral pH, the sugarcane bagasse was then bleached with 5% (*v*/*v*) NaClO at 60 °C for 1 h to give white and fine fibers. The reaction mixture was then filtered using gauze cloth and washed with distilled water until a neutral pH was reached. The obtained fibers were subsequently hydrolyzed with 0.5 M sulfuric acid at 70 °C for 1 h to break the long-chain cellulose fibers. Both bleaching and hydrolyzing steps were stirred continuously at 700 rpm. Finally, the suspension was filtered and washed with distilled water until the pH became neutral. The extracted cellulose was dried in a vacuum oven at 40 °C before its use as a substrate for the preparation of the SBG films and particles.

### 2.3. Extraction of Cellulose from Rice Straw

The rice straw used as raw material for cellulose extraction was prepared by following the previous report [27] with some modifications. Since rice straw contained high silica contents, cellulose extraction from rice straw employed harsh chemical conditions using high concentrations of strong acid and base. First, the rice straw was dewaxed like the sugarcane bagasse. The cellulose extraction process started with the hydrolysis of the dewaxed rice straw (1 g/10 mL) with 15% HCl (*v*/*v*) at 90 °C for 2 h. The hydrolyzed sample was filtered and washed several times with distilled water until the pH became neutral. After that, the sample was boiled in an alkaline solution with 20% (*w*/*v*) NaOH for 2 h at 90 °C to remove lignin and hemicellulose. After the alkaline treatment, the sample was washed continuously with distilled water and then bleached with 15% (*v*/*v*) NaClO for 2 h at 90 °C. Finally, the delignified residue or cellulose was filtered and then washed with distilled water until the pH turned neutral. The extracted cellulose was kept in a vacuum oven at 40 °C before use as a substrate for the preparation of the RS films and particles.

### 2.4. Preparation of the Cellulose Films

The extracted cellulose (1 g) from the sugarcane bagasse or the rice straw was suspended in 10 mL of distilled water. The mixture was stirred constantly for 1 h to form a homogeneous suspension before casting on the 9 cm diameter polystyrene Petri dishes. The suspension was left to dry at room temperature for 3 days. The films of the extracted cellulose mixed with 2.76% (*w*/*v*) glycerol were also prepared for comparison. The prepared cellulose films were peeled off from the Petri dishes and stored in a desiccator for further characterization.

### 2.5. Preparation of the Cellulose Particles

The cellulose particles were prepared by the water-in-oil (W/O) emulsification–diffusion method [39]. This method relies on the polarity index difference between the 2 phases. Ethyl acetate (polarity index = 4.4) was chosen as it is significantly less polar than water (polarity index = 10.2). When water from the cellulose matrix undergoes diffusion into ethyl acetate, it induces cellulose fibers to aggregate, leading to the formation of particles. In the process, the extracted cellulose suspension (1 mL) was used as a water (W) phase. Ethyl acetate (100 mL) was used as the oil (O) phase. The stirring speed was adjusted in the range of 500 to 700 rpm. In brief, ethyl acetate in the beaker was stirred rigorously on the magnetic stirrer apparatus. The cellulose solution (1 mL) was slowly added dropwise into the ethyl acetate while stirring continued for 30 min. To prevent the evaporation of the solvent during the emulsification and diffusion processes, the beaker was covered with aluminum foil. The particles were collected by centrifugation and then dried in a vacuum oven at room temperature until the solvent was entirely evaporated.

### 2.6. Characterization of the Films

The FTIR analysis of the films was performed using an FT-IR spectrophotometer (Invenio-S, Bruker, Karlsruhe, Germany) equipped with the ATR accessory. Each spectrum was recorded for 64 scans in the scan range of 4000 cm^−1^ to 400 cm^−1^ with a resolution of 4 cm^−1^.

The mechanical properties of films were evaluated using the tensile testing machine, following the ASTM D638 testing method. The film samples were cut into rectangular pieces (200 mm × 50 mm) and then fixed to the machine with tensile grips. The testing speed used was 2 mm/min at room temperature. The process was controlled and monitored by computer. Tensile strength (MPa) and elongation at break were obtained from the stress–strain curve. Five specimens of each were examined for mechanical changes.

The thermal decompositions of the sample films were determined using a thermogravimetric analyzer (TGA, SDT Q600, TA Instruments, New Castle, DE, USA). Each sample was heated from 50 °C to 600 °C at the rate of 20 °C/min under a nitrogen gas flow.

Phase morphology of the films and particles was examined using a scanning electron microscope (SEM, JSM-6460LV, JEOL, Tokyo, Japan). The prepared films were immersed in liquid nitrogen and cryogenically fractured. The samples were coated by gold sputtering before the SEM analysis at 15 kV.

The transparency of the constructed films was determined using a UV-Vis spectrophotometer (Lambda 25, Perkin Elmer, MA, USA), as previously described [40]. Briefly, the films were cut into rectangular pieces and placed directly in the spectrophotometer cell. Then, the percentage transmittance of light at 660 nm through each film was measured in triplicate to calculate the average film transparency.

The water susceptibility of the cellulose films was tested according to that previously reported [41]. The films were cut into a rectangular shape (1 cm × 2 cm) and dried at 100 °C until reaching constant weight (W_o_). Each film sample was placed into a test tube containing 10 mL distilled water and then left at room temperature for 24 h. After the period of 1, 3, 5, and 7 days, the nonsolubilized films were dried in an oven for 24 h and the final weight (W_f_) was measured. The measurements were conducted in triplicate for each time point. The results were reported as average values of % water susceptibility with standard deviation. The water susceptibility (%) values were calculated using the following Equation (1).

Water susceptibility (%) = [(W_o_ − W_f_)/W_o_] × 100 (1)

The moisture content of the cellulose films was assessed gravimetrically by determining the weight loss of the films, as previously described [41]. The obtained cellulose films, with a size of 1 × 2 cm^2^, were weighed before and after drying in an oven at 100 °C for 24 h to obtain a constant weight. The moisture content (%) of each film was determined by applying the following Equation (2):

Moisture content (%) = [(W_i_ − W_f_)/W_i_] × 100 (2)

where W_i_ and W_f_ are the weights of the samples before and after drying, respectively. Three replications of each film were performed to calculate the average values of moisture content. The results were reported as the average values of moisture content with standard deviations.

## 3. Results and Discussion

### 3.1. Extraction Yield of Cellulose

In this work, the extraction yields of cellulose from sugarcane bagasse and rice straw were 18.62 ± 1.24 and 7.81 ± 0.97 (%), respectively. The extracted cellulose from sugarcane bagasse in this work was obtained in higher content than the extracted cellulose from apple and kale pomaces previously reported [19]. However, it was about threefold lower than the yield of the microcrystalline cellulose (55%) extracted from sugarcane bagasse using a five-step process of alkaline peroxide and hydrolysis treatments [20,37]. Our lower extraction yield might be explained by the fact that the different parts of the material were used; the hard shell of sugarcane has higher cellulose content than the soft inner part. The cellulose content obtained from rice straw was also found to have a lower content than the total cellulose reported by previous studies [28,29]. The observed low cellulose yield can be attributed to the loss of cellulose fibers during the filtering and washing steps. This phenomenon can be ascribed to the micro/nanosized nature of the cellulose fibers obtained under the current working conditions. However, the variable cellulose content might be from plant varieties [18] and the chemicals and extraction methods used [19]. The removal efficiency of lignin content by the intense bleaching step could result in the reduction of the yield of cellulose. In addition, bleaching chemicals could destroy hydrogen bonds, resulting in the enhanced hydration ability of cellulose [19]. Strong acids like H_2_SO_4_ (sulfuric acid) and HCl (hydrochloric acid) are commonly used in cellulose extraction processes because they have the capability to break the glycosidic bonds present in the cellulose structure. This hydrolysis of the glycosidic bonds enables the separation of cellulose from other components in the raw materials. The use of sulfuric acid in the hydrolysis step has several disadvantages including low process yields [42]. Additionally, the obtained cellulose yield was also influenced by several other factors including acid concentration, time, temperature, and the ratio of acid to cellulose [20].

### 3.2. Transparency and Mechanical Properties of the Films

**Figure 1:**
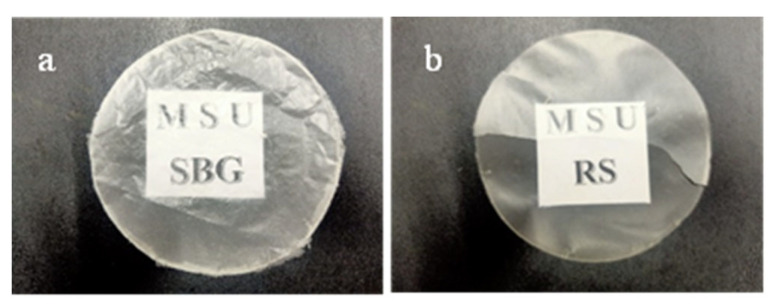
Macroscopic appearance of the native cellulose films extracted from sugarcane bagasse (**a**) and rice straw (**b**).

Transparency is one of the relevant properties of films, particularly for food packaging applications [41]. It allows for visualization of the internal container. Figure 1 shows the macroscopic aspect of the films prepared from SBG and RS cellulose. Both cellulose films are white and display homogeneous surfaces with high optical transmittance without holes. Compared to the native films, glycerol-mixed cellulose films exhibited a slight decrease in their transparency by about 13% and 5.6% for SBG and RS films, respectively (Table 1). The decreased transparency could be attributed to the hydrogen-bond interaction between hydroxyl groups of glycerol and cellulose, which promote the formation of the more crystalline part. Both SBG and RS films exhibited higher transparency than those cellulose films from apple and kale pomaces [18]. The transparency of the film depends on the size of the cellulose fibers. Small and short fibers contribute to a thin and transparent film, while longer cellulose fibers would lead to the production of thick and opaque films. The SBG film (Figure 1a) is milkier white than the RS film (Figure 1b) but has a lower % of light transmittance (Table 1). Regarding texture, the RS film is more brittle than the other film, possibly due to the high concentration of chemicals used in the cellulose extraction from rice straw. However, overall, the extracted cellulose from both SBG and RS was able to form transparent films, which is satisfactory considering the simplicity of the extraction process and the use of only common chemicals. Thus, SBG and RS cellulose are promising materials for film development and further investigations would be beneficial for improving their film properties.

Table 2 shows the mechanical test of the films. The results indicated that RS had the highest tensile strength (3.81 MPa), as well as Young’s modulus (21.58 MPa), but had the lowest elongation at break (1.8%). On the other hand, the native SBG film had lower tensile strength and Young’s modulus than the SBG film, approximately 13.6 and 10.9%. However, it has a higher elongation at break than RS, at about 27%. Additionally, the glycerol-mixed film resulted in a decrease in the mechanical strength of the films, both SBG and RS. The obtained results exhibited a similar trend for thermal stability. This was due to the glycerol-involved plasticization of the film texture and increased flexibility. This variation of mechanical properties was reflected by different factors including condition, additive substances, and instruments.

### 3.3. Morphological Observation

**Figure 2:**
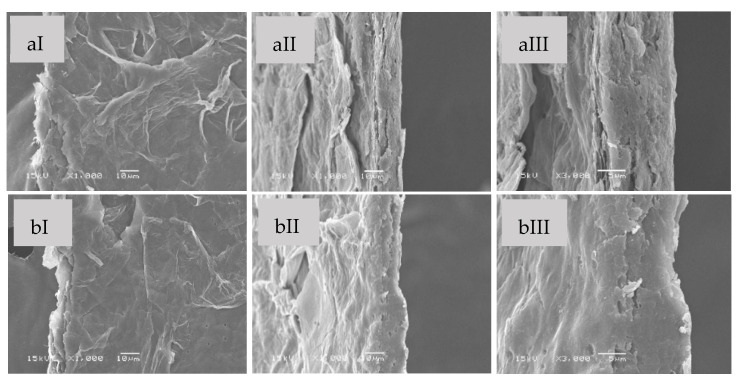
SEM images of the native SBG film (**a**) and the SBG mixed glycerol film (**b**); surfaces (I) and cross-sections with different magnifications (II = 1000× and III = 3000×).

**Figure 3:**
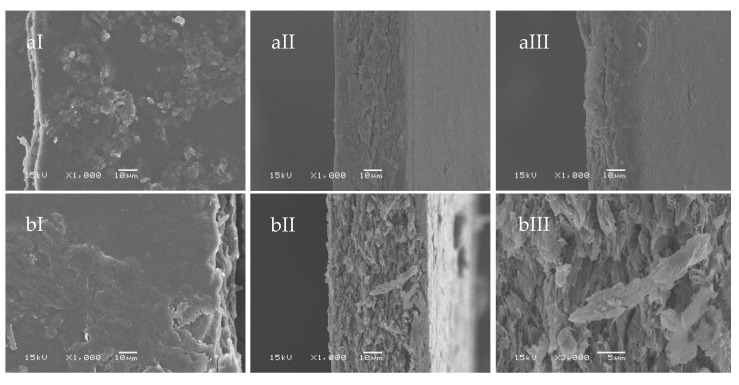
SEM images of the native RS film (**a**) and the RS mixed glycerol film (**b**); surfaces (I) and cross-sections with different magnifications (II = 1000× and III = 3000×).

The morphology of the prepared SBG and RS films were observed under SEM, as shown in Figure 2 and Figure 3, respectively. The native SBG film (Figure 2a) exhibited flat and thin fibers embedded on the film surfaces (Figure 2aI). The cellulose fibers were spread out and inserted into the film texture, resulting in a rough surface and nonwoven texture. This character was caused by the un-uniform shape of cellulose fibers. In the cross-sections, the SBG film was smooth in texture without phase separation, even at high magnification (Figure 2aIII). The morphology of the SBG mixed glycerol film (Figure 2b) had a looser cross-section texture than the SBG native film. The small and short fibers were well embedded into the film texture and merged slightly looser than that of the native film (Figure 2bI). This indicated that glycerol was dispersed in the cellulose films and increased the free volume in the film texture [43]. Moreover, the homogeneous texture of the films might be caused by the physical interaction among the glucose subunits as well as the chemical bonds between glucose and glycerol [37]. As shown in Figure 3, the RS native film (Figure 3a) had rough surfaces with a homogeneous texture. Some cellulose fibers appeared as small pieces with various non-uniform shapes. These small pieces allowed the fibers to be packed closer together, resulting in a dense texture and making the surfaces rougher than the SBG film. The unorganized arrangement of cellulose fibers might contribute to the decrease in the mechanical strength of the film. The cross-section of the RS film illustrated some grooves, clearly observed at high magnification (Figure 3aIII). The RS film surface (Figure 3bI) became smoother with the presence of glycerol, suggesting the interaction between glycerol and cellulose fibers. This agreed with previous reports suggesting that cellulose and glycerol formed H-bonds together [44,45]. At the cross-section, the film’s fracture and some grooves at the edge area were observed in the RS mixed glycerol film (Figure 3bII,III). It could be that glycerol enhanced the morphological changes inside the film texture, such as the formation of pores and grooves due to the water-bound glycerol evaporation [46]. From SEM micrographs, the RS films show smaller fibers on the surfaces compared to the SBG film. This indicated the effect of the high concentration of NaOH and HCl used for the extraction of cellulose from the rice straw. As discussed in the previous section, high concentrations of chemicals for pretreatment and bleaching could significantly affect the fiber dimension and damage cellulose fibers [19]. Therefore, the long and intact cellulose fibers rarely appeared in the film texture.

### 3.4. FTIR Analysis

**Figure 4:**
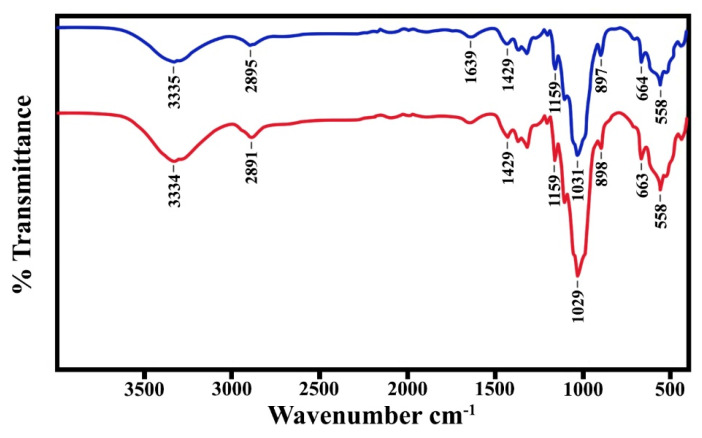
ATR-FTIR spectra of SBG film (upper) and SBG mixed glycerol (lower).

**Figure 5:**
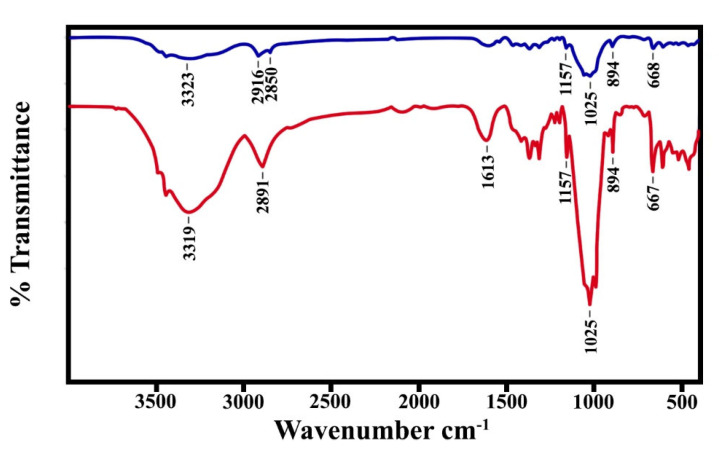
ATR-FTIR spectra of RS film (upper) and RS mixed glycerol (lower).

The functional groups of the prepared films were analyzed by FTIR spectroscopy. The FTIR spectra of the SBG films are presented in Figure 4. The typical bands at 3334, 2890–2895, 1429, 1030, and 897 cm^−1^, represent the O-H stretching, C-H stretching, C-H vibration, C-O-C pyranose ring skeleton, and β-(1-4)-glycosidic bond of cellulose, respectively [21,47,48]. The bands at 1159 and 1643 (1639) cm^−1^ were assigned to the arabinoxylan and C-O ester groups of hemicellulose, respectively [46]. However, the bands at 1159 cm^−1^ (C-O-C stretching; C-O stretching) are also associated with glycerol in the films [49,50,51]. Additionally, the C-O stretching of the hemicellulose peak should have appeared at about 1730 cm^−1^ [52]. With the addition of glycerol, the hydroxyl group increased, the -OH stretching peak at 3335 cm^−1^ and the peaks at 2916 and 2850 cm^−1^ (C-H stretching) gradually increased. The outstanding bands of O-H and C-H stretching were observed in the glycerol-mixed SBG films. This means that the hydroxyl group of glycerol had H-bond interactions with plenty of hydroxyl groups in cellulose [18]. The FTIR spectra of the RS film are shown in Figure 5. Overall, most absorption bands of the RS film have the same wavenumber as those that appeared in the SBG film, except the small peak at 1540 cm^−1^ which represents the C=C group of the lignin aromatic network [52]. The peaks at the C-H region (2920–2895 cm^−1^) in the films without glycerol are typical peaks from aromatic residues in lignin. This means that the lignin was not fully removed from the fiber by treatment with alkali and bleaching.

### 3.5. Thermal Stability

**Figure 6:**
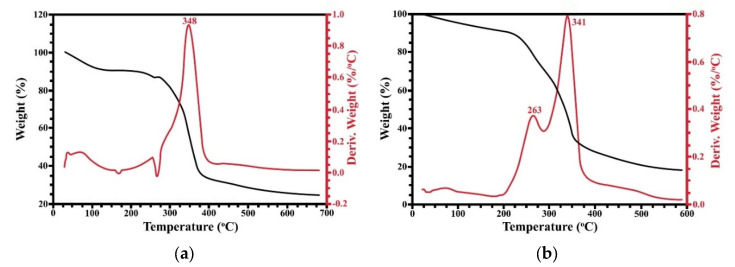
TG/DTG thermograms of SBG film (**a**) and SBG-mixed glycerol film (**b**).

**Figure 7:**
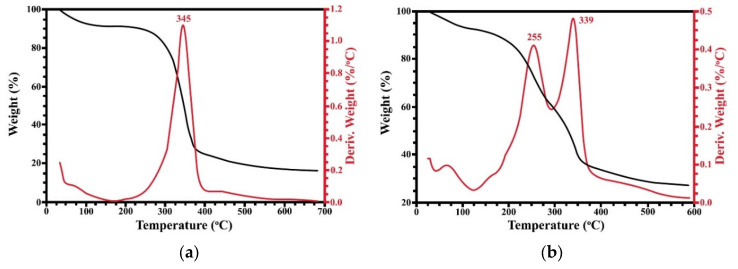
TG/DTG thermograms of RS film (**a**) and RS-mixed glycerol film (**b**).

The thermal stability of the films was investigated by thermogravimetric analysis. Figure 6 and Figure 7 show the mass loss curves versus temperature and the derivative thermogravimetric (DTG) curves of the SBG and RS films, respectively. According to Figure 6, the native SBG film has at least two stages of mass loss. The first stage at lower than 100 °C is due to the evaporation of water in the films [47,53]. Water could interact with the hydroxyl groups of cellulose via hydrogen bonds. The second stage of mass loss occurred between 300 to 350 °C. This weight loss was the main degradation and was related to the degradation of cellulose [54]. In the case of the glycerol-mixed SBG films, three degradation stages were observed. There was an initial state of mass loss up to 100 °C, which was caused by the water loss. The second stage of mass loss appeared at 263 °C, which was the loss of glycerol evaporation, and appeared with a small shoulder peak. However, this region might be the mass loss of hemicellulose and, partially, lignin [52,55], which remained in the extracted cellulose. The maximum decomposition temperature or T*_d,max_* of the native and glycerol-mixed SBG films were 348 and 341 °C, respectively. This result indicated that the addition of glycerol resulted in a decrease in the thermal stability of the SBG film. This was due to the addition of glycerol affecting the water absorption of the cellulose fibers. The absorbed water molecules would increase the distances between the cellulose chains. Thus, the interactions between the fibers would be interrupted and lead to the decrease in thermal stability.

The native RS film (Figure 7a) has two stages of mass loss. The initial stage was water loss which occurred at lower than 100 °C. The second stage of mass loss was 345 °C which was expected to be the mass loss of cellulose [56,57]. Similar to the SBG film, the glycerol-mixed RS film showed three stages of mass loss. However, the maximum decomposition temperature of the RS film was slightly decreased by the addition of glycerol. The T*_d,max_* of the glycerol-mixed RS films was 339 °C. The RS film with glycerol started to degrade rapidly earlier than the native film. This might be explained by the fact that glycerol could be dispersed throughout the film and decrease the crystallinity of the cellulose [44,58,59]. As a result, the T*_d,max_* of the RS-mixed glycerol was lower than the native RS film. At the end of the measurement, the weight of both the native and glycerol-mixed of the SBG and RS films remained about 20%. This remaining weight was carbon charcoal, which was not decomposed. This value was also varied by sources or material characteristics, as well as the interaction between the components [44,60]. Compared to the sausage fibers, the cellulose fibers from sugarcane bagasse and rice straw have higher thermal stability [52]. However, the results are consistent with the previously reported thermal stability of other cellulose from sugarcane bagasse [37] and rice straw [30].

### 3.6. Water Susceptibility

The moisture content in the prepared films was shown in Table 1. The RS films show significantly higher moisture content than the SBG films. When comparing among the native films and RS film, it is well known that natural polysaccharides have poor water-vapor barriers due to their inherent hydrophilic property. The films with glycerol have higher moisture content than their corresponding native films. This may be due to the interaction between the water molecules and the hydrophilic part in glycerol hydroxyl groups [59,61], which leads to an increase in the hydrophilicity of the film surfaces. The native RS film showed a water susceptibility of 15%; the water susceptibility of the native SBG film was 8% after 7 days. Compared with the native films, the films with glycerol exhibited higher percent water susceptibilities of 32% and 40% after a 7-day test for SBG and RS mixed films, respectively. The results indicated that the RS films have higher susceptibility to water than the SBG films. This might be attributed to the harsh conditions used for cellulose extraction, which broke the cellulose chains and chemical interactions.

### 3.7. Morphological Observation of Particles

**Figure 8:**
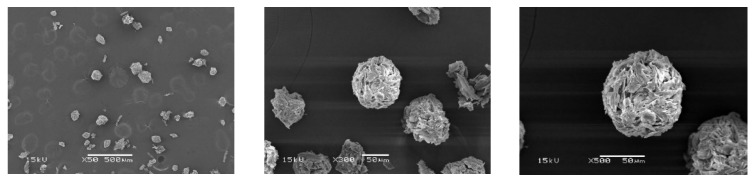
SEM Images of SBG Particles at Different Magnifications.

**Figure 9:**
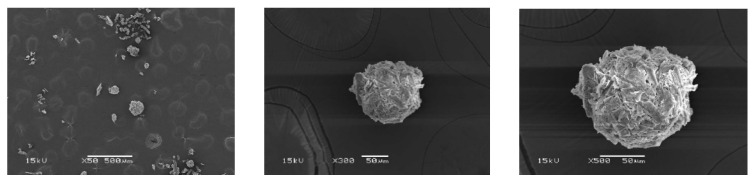
SEM Images of RS Particles at Different Magnifications.

In this work, the ratio of water and oil phases (W:O) used in the preparation of the particles was 0.1:100. Both SBG (Figure 8) and RS (Figure 9) particles did not have complete spherical shapes, as revealed by the SEM images. The particles were formed by the physical interaction between hydrolyzed cellulose chains. Comparing SBG and RS cellulose, the SBG had shorter cellulose chains than the RS, resulting in a denser network in the texture of the particles. In addition, the SBG particles appeared more spherical than the RS particles. Generally, spherical particles could be easily prepared from polymer solutions. However, the extracted cellulose in this work was obtained as micro/nano-size fibers and suspended in water as suspension. The fiber length was an important factor in the formation of spherical particles. The suitable extraction conditions for cellulose from sugarcane bagasse and rice straw that would achieve sufficiently small chains to aggregate homogeneously for particle formation have never been reported. Nevertheless, the obtained results revealed that the extracted cellulose from SBG and RS could form into particles successfully. Recently, cellulose-based particles have been developed for various applications, especially in drug delivery systems [62,63,64]. Although more research is still needed to synthesize consistent and complete spherical particles, this work showed promising particle formation using the cellulose extracted from agricultural waste using a simple protocol.

## 4. Conclusions

Our focus was on utilizing sugarcane bagasse and rice straw as raw materials to develop higher-value products like films and particles, aiming to provide sustainable alternatives to plastic packaging and to enable drug encapsulation in medical applications. The cellulose fibers were successfully extracted from sugarcane bagasse and rice straw by simple alkali (NaOH) treatment, NaClO oxidation, and acid hydrolysis. The extracted cellulose could be used as material for the preparation of biodegradable films and particles. The prepared films showed high light transmittance and transparency. They were examined for surface morphology and FTIR spectral patterns as well as thermal stabilities. The extraction of cellulose from rice straw involved the use of stronger chemical treatments compared to the process for sugarcane bagasse. The resulting RS films had higher light transmittance, water dissolubility percentage, and tensile strength, but lower elongation and thermal stability compared to the SBG films. The RS films exhibited high moisture contents and rapidly disintegrated in water. The addition of glycerol significantly affected the moisture contents, water susceptibility, mechanical properties, and the T*_d,max_* values of both SBG and RS films. Cellulose particles with almost spherical shapes were also prepared. Therefore, the obtained results would be further used as a guideline for cellulose-particle improvement. These results demonstrated that agricultural waste such as sugarcane bagasse and rice straw in Thailand could serve as renewable, sustainable, and low-cost sources of cellulose. The extracted cellulose from these wastes can be used as materials to prepare biodegradable high-value-added films and particles for further applications.

## Figures and Tables

**Table 1:** Transparency and Moisture Content of the SBG and RS Films.

Samples	T660 (%)	Moisture Content (%)	Water Susceptibility (%)
SBG Native film SBG + glycerol	91.53 ± 0.42	4.43 ± 0.31	8 ± 0.13
	79.70 ± 1.71	6.27 ± 0.39	32 ± 0.25
RS Native film RS + glycerol	97.30 ± 3.48	14.49 ± 2.76	15 ± 0.45
	91.83 ± 3.64	15.80 ± 1.38	40 ± 0.67

**Table 2:** Mechanical properties of the SBG and RS Films.

Samples	Force @ Peak (N)	Tensile Stress (MPa)	Elongation @ Break (%)	Young’s Modulus (MPa)
SBG	15.05	3.29	2.47	19.23
SBG/Glycerol	17.47	3.10	2.70	16.22
RS	16.61	3.81	1.80	21.58
RS/Glycerol	15.70	3.64	2.23	19.86
Maximum	17.47	3.81	2.80	21.86
Minimum	15.05	3.10	2.47	16.58
Mean	16.21	3.46	2.62	19.22

## Data Availability

Not applicable.
